# A Comparative Study of Burden of Care, Anxiety, and Well-Being Among Family Caregivers of Elderly with Dementia: Evidence from Kuwait

**DOI:** 10.3390/healthcare13141767

**Published:** 2025-07-21

**Authors:** Fahad Manee, Musaed Z Alnaser, Ali Alqattan, Sheikha Almutairi, Hessa Maqtouf

**Affiliations:** 1 Occupational Therapy Department, Faculty of Allied Health Sciences, Kuwait University, P.O. Box 31470, Sulaibekhat 90805, Kuwait; musaed.alnaser@ku.edu.kw; 2 Kuwait Ministry of Health, Mubarak Al-Kabeer Hospital, P.O. Box 47060, Jabriya 43755, Kuwait; dr.alialqattan@hotmail.co.uk; 3 Occupational Therapy Department, Physical Medicine and Rehabilitation Hospital, Ministry of Health, P.O. Box 4079, Sulaibekhat 13041, Kuwait; sheikhaalhelan@gmail.com (S.A.); hessa.maqtouf@gmail.com (H.M.)

**Keywords:** dementia, mental health, dementia caregivers, burden of care, Kuwait

## Abstract

**Background and Objectives:** Caring for an individual with dementia encompasses many challenges. This can lead to increased burden, anxiety, and mental health issues among those taking care of them. Limited research exists investigating the care of people with dementia in Kuwait, particularly regarding the mental health of caregivers. There is a need to understand the impact of caregiver burden in this population. This study aimed to assess the level of burden of care, depression, anxiety, and well-being among caregivers of the elderly with dementia in Kuwait. **Methods:** This study used a descriptive and cross-sectional design. To measure the burden of care, depression, anxiety, and well-being of the caregivers, we utilized the Zarit Burden Interview, the Hospital Anxiety and Depression Scale, and the World Health Organization-Five Well-Being Index. A sensitivity analysis was conducted to compare the results of the parametric and non-parametric methods. **Results:** This study included 180 (65%) caregivers for the elderly with dementia and 98 (35%) without dementia. The descriptive statistics showed that caregivers for the elderly with dementia and caregivers for the elderly without dementia experienced moderate burden (17.21 ± 9.09 and 14.51 ± 8.08, respectively), borderline abnormal anxiety (9.92 ± 5.15 and 8.61 ± 4.79, respectively), borderline abnormal depression (8.69 ± 4.35 and 8.06 ± 4.24, respectively), and low mental health well-being (54.40 ± 25.10 and 58.90 ± 23.42, respectively). The *t*-test of independent samples and Mann–Whitney U test results showed that the burden and anxiety in the caregivers for the elderly with dementia group were statistically significantly higher than those in the caregivers for the elderly without dementia group (*p* = 0.015 and *p* = 0.039; *p* = 0.026 and *p* = 0.027, respectively). The ANOVA test and Kruskal–Wallis test revealed that the caregivers for the elderly with dementia group had statistically significant differences in burden (*p* < 0.001; *p* < 0.001), anxiety (*p* = 0.048; *p* = 0.043), depression (*p* = 0.017; *p* = 0.009), and mental health well-being (*p* = 0.001; *p* = 0.002) scores across various durations of care. The multiple linear regression showed that caregiving was a significant predictor of burden of care and anxiety, indicating that caregivers of the elderly with dementia experienced a higher burden of care than those caring for the elderly without dementia. In addition, confounders with significant influence were duration of care (*p* < 0.001), education level (*p* = 0.002), employment status (*p* = 0.008), and gender (*p* = 0.02). **Conclusions:** Family caregivers experienced significant levels of burden of care and anxiety when caring for the elderly with dementia. A multidimensional holistic approach is needed to provide family caregivers of the elderly with dementia with valuable interventions.

## 1. Introduction

The global aging population is on an unprecedented rise, with the World Health Organization (WHO) projecting that the number of individuals aged 60 years and above will surpass two billion by 2050. While indicating longer life expectancies, this demographic shift also increases age-related health issues. Among these, dementia emerges as a prevalent concern [1,2], with estimations suggesting that the number of affected individuals may reach 75 million by 2030 [3]. The most common cause of dementia, in 60% to 80% of all cases, is Alzheimer’s disease [4]. 

Dementia, a neurological disorder, intricately impacts various cognitive functions, including memory, thinking, orientation, comprehension, calculation, learning, language, and judgment. The cognitive decline is associated with difficulties controlling emotions, interacting with others, and being self-motivated. Common signs and symptoms of dementia in the early stage include forgetfulness, losing track of time, and becoming lost in familiar places. The middle stage of dementia includes becoming confused while at home, having increasing difficulty with communication, and needing help with personal care and daily activities. The late stage includes becoming unaware of the time and place, increased need for assistance with self-care and daily activities, difficulty walking, and experiencing behavior changes such as aggression [5]. The spectrum of symptoms, ranging from simple forgetfulness to profound behavioral changes such as aggression, places a considerable burden on those caring for individuals with dementia and underscores the crucial role caregivers play in managing and mitigating the impact of dementia [6].

Family members often find themselves assuming the role of caregivers when their relatives are affected by dementia. A sense of duty, spiritual obligation, and social expectations can drive the role of caregiving. While caregivers play a crucial role in supporting people with dementia, the gradual challenges in orientation, communication, self-care, and daily activities necessitate an increasing level of care [7]. Unfortunately, the provision of this support comes at a cost to caregivers, manifesting as a burden, depression, anxiety, compromised and diminished mental health well-being, and restricted participation in leisure and social activities [8,9]. 

The average length of dementia from onset to death is approximately 4.6 years [3]. Studies’ findings indicate that family caregivers of people with dementia experience abnormal levels of burden, anxiety, and depression [10,11]. The term “caregiver burden” succinctly captures the multifaceted impact of dementia on those providing care. It represents a state of physical, emotional, and mental fatigue, often manifesting as depression, anguish, worry, and other symptoms [12]. Further, caregivers may experience psychological distress, including somatization, obsessive–compulsive behavior, interpersonal sensitivity, anxiety, hostility, and paranoid ideation [13], coupled with physical symptoms such as sleep disturbances [14]. These symptoms can become increasingly prevalent as the level of dependency in individuals with dementia rises over time [13,14]. Moreover, caregivers of the elderly with dementia exhibit more severe burden, anxiety, stress, and depressive symptoms compared to caregivers of patients without dementia [15]. Research studies have examined the relationship between family caregivers of people with dementia and the risk of anxiety, depression, and burden, mainly in Western countries and cultures [7]. The primary objective of this study was to assess and compare the level of burden of care, depression, anxiety, and mental health well-being among caregivers of the elderly with dementia (CEWDs) to that of caregivers without dementia (CEWoDs). Additionally, this study aims to shed light on the challenges faced by CEWDs and CEWoDs in Kuwait, a topic of significant importance in the context of the global aging population. We hypothesized that the CEWDs group would experience higher levels of burden, depression, and anxiety, and lower mental health well-being.

## 2. Methods

### 2.1. Study Design

This study employed a descriptive and cross-sectional design to examine variables related to the burden of care, anxiety, depressive symptoms, and mental health well-being among caregivers of the elderly with and without dementia. The cross-sectional design helps describe the target population’s characteristics and establishes introductory evidence in formulating a future expanded study [16]. We compared variables of burden of care, anxiety, depressive symptoms, and mental health well-being to caregivers’ experiences. 

### 2.2. Participants

The target population consisted of male and female caregivers of the elderly with chronic health conditions (with and without dementia). Inclusion criteria included caregivers aged 21 years and above who had been caring for an elderly family member for over six months. The individuals being cared for were over 60 years old and had chronic health conditions and the presence or absence of dementia. Caregivers were excluded if they were below 21 years of age. The patients were excluded if they were less than 60 years old, without chronic conditions, and with communication problems or with a score < 7 as screened by the Short Portable Mental Status Questionnaire (SPMSQ), indicating severe cognitive affection [17].

An a priori power analysis was conducted to determine the minimum sample size required to test the study hypothesis using parametric and non-parametric analyses. G*Power version 3.1.9.7 [18] was used to determine the minimum sample size. The analysis assumed an unequal group allocation ratio of 2:1, with the expected distribution of participants in the dementia and non-dementia caregiver groups. Results indicated that the required sample size to achieve 95% power for detecting a medium effect, at a significance criterion of α = 0.05, was N = 236 for the *t*-test for independent samples and N = 280 for the ANOVA. A medium effect size was defined as Cohen’s d = 0.5.

For the non-parametric analysis, the results indicated that the required sample size to achieve 95% power for detecting a medium effect, at a significance criterion of α = 0.05, was N = 274 for the Mann–Whitney test, N = 320 for the Kruskal–Wallis test, and N = 191 for the chi-square test. For the Mann–Whitney test, the effect sizes are calculated using the formula r = Z/√N, where Z is the standardized test statistic and N is the total number of observations. According to Cohen’s guidelines, a medium effect is represented by a value of 0.30. For the Kruskal–Wallis test, the effect sizes are calculated using the formula ղ^2^ = (H − k + 1)/(n − k), where H is the Kruskal–Wallis test statistic, k is the number of groups, and n is the total sample size. Then, the ղ^2^ is converted into Cohen’s f (√ղ^2^/1 − ղ^2^). According to Cohen’s guidelines, 0.25 represents a medium effect size. For the chi-square test, the effect sizes are calculated using the formula ω = √X^2^/N, where X^2^ is the chi-square statistic and N is the total sample size. According to Cohen’s guidelines, 0.30 represents a medium effect.

### 2.3. Instrumentation

#### 2.3.1. Demographic Information

Comprehensive demographic questions were used to collect information from the caregivers, including age, gender, kin relationship, educational level, employment, and the level of assistance from others. Demographic questions were also designed to collect information about elderly individuals, including age, gender, and dementia status. 

#### 2.3.2. The Zarit Burden Interview

The Zarit Burden Interview (ZBI-12), a standardized and validated screening tool, was employed to measure the burden associated with caring for individuals with advanced illnesses, such as dementia [19,20]. The tool has proven reliability for the subjective assessment of the burden on caregivers of dementia patients in different settings [21]. It has been demonstrated to possess good internal consistency, with a Cronbach’s α of 0.90 [22]. 

The short version of the ZBI comprises 12 items, divided into two domains: personal strain and role strain. Each question is scored on a five-point Likert scale from 0 to 4 (never to almost always). High scores indicate a greater sense of burden. The range of the summed score is 0–48. The ZBI-12 underwent validation, and its psychometric properties were assessed for Arabic-speaking individuals, in addition to internal consistency with Cronbach’s α of 0.77 [23].

#### 2.3.3. The Hospital Anxiety and Depression Scale

The Hospital Anxiety and Depression Scale (HADS) has been widely used and validated for measuring anxiety and depression cases [24]. The HADS anxiety and depression subscales have been shown to possess good internal consistency and reliability, with Cronbach’s α ranging between 0.67 and 0.90 [25].

The HADS is a 14-item, non-symptom-related, self-assessment scale designed to detect perceived levels of anxiety and depression among nonpsychiatric patients. All items are equally weighted on a 4-point scale, where 0 reflects the positive extreme and 3 reflects the negative extreme of the scale [26]. The HADS contains two 7-item anxiety and depression subscales, which are summed to obtain scores ranging from 0 to 21. For each subscale, the scores are categorized as normal (0–7 points), mild (8–10 points), moderate (11–14), and severe (11–21 points); scores ≥ 8 are considered elevated. The HADS has been validated in many languages, countries, and settings. The Arabic version, with a Cronbach’s α of 0.83, was employed for this study [27].

#### 2.3.4. The WHO-5 Well-Being Index

The WHO-5 Well-Being Index is a psychometrically sound measure [28,29]. It is a brief questionnaire consisting of 5 non-invasive questions, measuring subjective mental health well-being on a 6-point Likert scale ranging from 0 = “at no time” to 5 = “all of the time” [30]. The raw score, ranging from 0 to 25, was multiplied by 4 to yield a final score ranging from 0 to 100, where 0 represents the worst and 100 the best mental health well-being. The WHO-5 has been found to have adequate construct validity and has been translated into many languages [28]. It has been demonstrated to possess good internal consistency, with a Cronbach’s α of 0.92 [31]. The Arabic version, with a Cronbach’s α of 0.70, was adapted by Ohaeri and Awadalla [32] and utilized in this study.

### 2.4. Procedure

Ethical approval for this study was obtained from the Institutional Review Board and Ethical Committee of the Health Sciences Center at Kuwait University and the Kuwait Ministry of Health (Ref/606, 2024). Caregivers were recruited from a local hospital’s list of caregivers/patients. A link to the instrument (demographic information, ZBI-12, HADS, and WHO-5) was sent to the caregivers through a social media application. Caregivers could choose between Arabic or English versions of the instrument. The online survey commenced with a statement outlining this study’s objective and a consent statement that participants had to sign before proceeding. Subsequently, participating caregivers completed the demographic questions, ZBI-12, HADS, and WHO-5 Well-Being Index. This systematic approach could ensure standardized data collection across all participants, contributing to the robustness of this study. 

### 2.5. Data Analysis

The Statistical Package for the Social Sciences (SPSS), version 26 [33], was employed for data analysis. Descriptive statistics, encompassing frequencies, means, standard deviations, and percentages, were used to summarize demographics and assessment outcomes. Additionally, a chi-square (χ^2^) test was used to determine the association between the CEWDs and CEWoDs groups and the demographic factors. There was homogeneity of variances between groups, as assessed by the Levene’s test for equality of variances (*p* > 0.05). Normality of the data was assessed using the Shapiro–Wilk test. The assumption of normality was not met for the burden of care (W(278) = 0.978, *p* < 0.001), anxiety (W(278) = 0.979, *p* < 0.001), depression (W(278) = 0.971, *p* < 0.001), and mental health well-being (W(278) = 0.977, *p* < 0.001). However, according to the Central Limit Theorem, the sampling distribution of the sample mean approaches a normal distribution when the sample size is sufficiently large, regardless of the population’s distribution [34]. Therefore, the sample size in this study (N = 278) was sufficiently large to permit the use of parametric analysis. Therefore, a *t*-test of independent samples was used to compare the two groups on the burden of care, depression, anxiety, and mental health well-being. An ANOVA test was used to compare the four assessments (ZBI-12, HADS (anxiety and depression), and WHO-5) on the duration of caring for the CEWDs and CEWoDs groups. In addition, due to the violations of normality, non-parametric equivalents, such as the Mann–Whitney U test and the Kruskal–Wallis test, were applied. Utilizing sensitivity analysis would allow assessing the robustness of the finding. 

Moreover, a multiple linear regression was conducted to examine whether caregiving for the elderly with dementia and without dementia predicts the caregiver burden, anxiety, depression, and mental health well-being, after adjusting for age, gender, education, employment, and duration of care. The threshold for significance was set at *p* < 0.05 [35]. 

## 3. Results

Table 1 presents demographic information about the participating caregivers. This study included 180 (65%) caregivers for the elderly with dementia (CEWDs) and 98 (35%) without dementia (CEWoDs), with an 87% response rate. The participants were not evenly distributed across the groups and in terms of demographic characteristics. However, most caregivers in the sample were female (204, 73%), married (162, 58%), and adult daughters (189, 68%) of the individuals receiving care (Table 1). The mean age of the CEWDs group was 48.71 ± 10.74 years, while the CEWoDs group was 42.81 ± 14.27 years. More than 80% of the two groups had been caregivers for over a year, with 8% (fourteen CEWDs and nine CEWoDs) caring for more than ten years. Approximately half of the CEWDs group (48%) performed all caring-related tasks, compared to 35% for the CEWoDs group. The *t*-test of independent samples results showed that the age of the CEWDs group (µ = 48.71 ± 10.74) was significantly higher than that of the CEWoDs group (µ = 42.81, SD = ±14.27) (t(276) = −3.887, *p* < 0.001, Cohen’s d = 0.488).

Moreover, the mean age of the elderly with dementia was 79.47 ± 8.13, whereas the mean age of the elderly without dementia was 75.24 ± 9.01, with a female majority (123 (68%) and 66 (67%), respectively). Most of the elderly with dementia were married (86; 48%), whereas most of the elderly without dementia were widowed (49; 50%). The chi-square test results showed an association between both elderly groups and marital status (*p* = 0.010); however, no association was found between both groups of elderly and gender (*p* = 0.866) (Table 2).

The internal consistency of the instruments was assessed using Cronbach’s alpha. All scales demonstrated acceptable reliability within the current sample (N = 268). The following were the results: α = 0.81 for the Zarit Burden Interview, α = 0.79 for the anxiety subscale of HADS, α = 0.82 for the depression subscale of HADS, and α = 0.76 for the WHO-5. The descriptive statistics showed that CEWDs and CEWoDs experienced moderate burden (µ = 17.21, SD = ±9.09 and µ = 14.51, SD = ±8.08, respectively), borderline abnormal anxiety (µ = 9.92, SD = ±5.15 and µ = 8.61, SD = ±4.79, respectively), borderline abnormal depression (8.69 ± 4.35 and 8.06 ± 4.24, respectively), and low mental health well-being (µ = 54.40, SD = ±25.10 and µ = 58.90, SD = ±23.42, respectively). Moreover, the severe burden was 38% among CEWDs compared to 24% among CEWoDs. Low mental health well-being was approximately similar between the two groups (59% and 53%, respectively). Abnormal anxiety and depression were 55% and 46% among the CEWDs group compared to 33% and 28% among the CEWoDs group (Table 3). Moreover, the *t*-test of independent samples and the Mann–Whitney U test results showed that the burden in the CEWDs group was statistically significantly higher than that in the CEWoDs group (t(276) = −2.454, *p* = 0.015, Cohen’s d = 0.31; U = 7400, *p* = 0.026, r = 0.13). Also, the anxiety in the CEWDs group was statistically significantly higher than that in the CEWoDs group (t(276) = −2.073, *p* = 0.039, Cohen’s d = 0.26; U = 7403, *p* = 0.027, r = 0.13). However, the results of the *t*-test of independent samples and the Mann–Whitney U test showed that there was no significant difference in mental health well-being (t = 1.461, *p* = 0.145; U = 7859.5, *p* = 0.133) and depression (t = −1.159, *p* = 0.247, U = 7857, *p* = 0.130) between CEWDs and CEWoDs (Table 3). Findings from the sensitivity analysis indicated that the results were robust to violations of the normality assumption. In addition, the chi-square test of independence revealed a significant association between the burden of care level (*p* = 0.034), anxiety level (*p* = 0.002), and depression level (*p* = 0.012) with the caregiver groups (dementia vs. no dementia). High levels were more likely to be reported by caregivers of the elderly with dementia. No association was found between the mental health well-being level (*p* = 0.644) and the caregiver groups (Table 3).

Moreover, among the CEWDs group, the ANOVA test and Kruskal–Wallis test showed that there was a statistically significant difference in burden (*p* < 0.001; *p* < 0.001), anxiety (*p* = 0.048; *p* = 0.043), depression (*p* = 0.017; *p* = 0.009), and mental health well-being (*p* = 0.001; *p* = 0.002) scores on the various durations of caring (<1, 1–5, 6–10, >10 years). Bonferroni-adjusted pairwise comparisons showed that the CEWDs group with 1–5, 6–10, and >10 years of caregiving had a significantly higher burden of care than with <1 year of caregiving (*p* < 0.001) (Table 4).

However, among the CEWoDs group, the ANOVA test and Kruskal–Wallis test showed statistically significant differences in burden (*p* = 0.004; *p* = 0.002) and depression (*p* = 0.021; *p* = 0.032) scores across different durations of caregiving. No statistically significant difference was found for mental health well-being scores (*p* = 0.260) across various durations of caregiving (Table 4). The only disagreement between the ANOVA test and the Kruskal–Wallis test was found on the anxiety variable (*p* = 0.057; *p* = 0.043). The Bonferroni-adjusted pairwise comparisons revealed significant differences only between <1 year and 1–5 years of caregiving for burden (adjusted *p* = 0.004) and depression (adjusted *p* = 0.030) among the CEWoDs group (Table 4).

Moreover, among the CEWDs group, the *t*-test of independent samples test showed statistically significant gender differences in anxiety (t(178) = 1.322, *p* = 0.002, Cohen’s d = 0.53), depression (t(178) = 2.022, *p* = 0.045, Cohen’s d = 0.34), and mental health well-being (t(178) = −2.068, *p* = 0.040, Cohen’s d = 0.35), with female caregivers reporting higher anxiety (µ = 10.62, SD = ±5.125), higher depression (µ = 9.08, SD = ±4.270), and lower mental health well-being (µ = 52.12, SD = ±25.951) compared to male caregivers (anxiety µ = 7.96, SD = ±4.782), depression µ = 7.60, SD = ±4.431, and well-being µ = 60.85, SD = ±21.523). No statistically significant gender differences were found among the CEWDs group for burden of care (*p* = 0.188). In addition, no statistically significant gender differences were found among the CEWoDs group for burden (*p* = 0.820), anxiety (*p* = 0.440), depression (*p* = 0.698), and mental health well-being (*p* = 0.865). 

For the multiple linear regression, the assumptions were tested.

1. The Shapiro–Wilk test (*p* < 0.001) showed that the data were not normally distributed. However, due to the large sample size, the assumption was considered to be met.
2. The correlations between the predictor variables were less than 0.7, so the variables were not multicollinear. In addition, collinearity statistics included tolerance > 0.2 and VIF < 5.
3. The standard residuals were within ±3. Therefore, no outliers were found.
4. Cock’s distances were <1, indicating the absence of outliers.

First, the multiple linear regression analysis was conducted to examine whether caregiving for the elderly with dementia and without dementia predicts caregiver burden, after adjusting for age, gender, education, employment, and duration of care. The overall model was statistically significant, F(5,271) = 7.065, *p* < 0.001, with an R^2^ = 0.134. Caregiving was a significant predictor of burden of care (B = −2.695, *p* = 0.015), indicating that caregivers of the elderly with dementia experienced a higher burden of care than those caring for the elderly without dementia. Additionally, caregivers’ duration of care (B = 2.627, *p* < 0.001), education (B = 1.938, *p* = 0.002), and employment (B = −1.289, *p* = 0.008) were also significant predictors, with greater duration of care, lower education, and being unemployed associated with higher burden of care levels.

Second, the multiple linear regression analysis was conducted to examine whether caregiving for the elderly with dementia and without dementia predicts caregiver anxiety, after adjusting for age, gender, education, employment, and duration of care. The overall model was statistically significant, F(5,271) = 2.748, *p* < 0.019, with an R^2^ = 0.063. Caregiving was a significant predictor of anxiety (B = −1.310, *p* = 0.039), indicating that caregivers of the elderly with dementia experienced higher anxiety than those caring for the elderly without dementia. Additionally, caregivers’ gender (B = 1.602, *p* < 0.020) and employment (B = −0.571, *p* = 0.048) were also significant predictors, with being a female and unemployed associated with higher anxiety levels. 

Third, the multiple linear regression analysis was conducted to examine whether caregiving for the elderly with dementia and without dementia predicts caregiver mental health well-being, after adjusting for age, gender, education, employment, and duration of care. The overall model was statistically significant, F(5,271) = 3.552, *p* < 0.004, with an R2 = 0.069. However, caregiving was not a significant predictor of mental health well-being (B = 4.498, *p* = 0.145), indicating that caregivers of the elderly with dementia did not experience higher mental health well-being than those caring for the elderly without dementia. Additionally, caregivers’ duration of care (B = −5.186, *p* < 0.004), education (B = −3.928, *p* = 0.028), and gender (B = −7.155, *p* = 0.031) were significant predictors, with greater duration of care, lower education, and being female associated with lower mental health well-being levels. 

Finally, the multiple linear regression analysis was conducted to examine whether caregiving for the elderly with dementia and without dementia predicts caregiver depression, after adjusting for age, gender, education, employment, and duration of care. The overall model was not statistically significant, F(5,271) = 2.111, *p* < 0.064, with an R^2^ = 0.042. Caregiving was not a significant predictor of depression (B = −0.628, *p* = 0.247), indicating that caregivers of the elderly with dementia did not experience depression more than those caring for the elderly without dementia. Additionally, caregivers’ duration of care (B = 0.777, *p* < 0.015) was a significant predictor, with greater duration of care associated with depression level.

## 4. Discussion

To the best of our knowledge, this study is the first to explore the effects of caregiving for individuals with dementia in Kuwait. This study’s findings showed that the participating caregivers for individuals with and without dementia in Kuwait predominantly experienced moderate to severe burden of care and borderline to abnormal anxiety and depression, in addition to low mental health well-being. Moreover, this study’s findings showed that CEWDs were significantly impacted by the burden of care and anxiety when compared to caring for the elderly without dementia. In addition, results revealed that CEWDs experienced higher levels of burden of care, anxiety, depression, and lower mental health well-being. 

The limited access to dementia resources, cultural norms that place a heavy burden on family caregivers, and a lack of awareness and support systems in Kuwait can negatively impact the quality of life for caregivers. This can potentially lead to increased burden, anxiety, and mental health problems and challenges faced by caregivers.

These findings are comparable to other studies conducted regionally and internationally [36,37,38,39,40]. Regionally, Abdelhalim et al. conducted a comparable study in Egypt among 141 CEWDs, which showed a substantial burden in providing care and elevated levels of despair and anxiety among caregivers of PWD. 

Internationally, Teahan et al. studied 2311 family caregivers of older people. About 20% of the care recipients had a diagnosis of dementia. The findings demonstrated significant differences in caregiver burden distribution, with family caregivers of people with dementia being significantly more likely to report moderate or high caregiver burden. Quinn et al. included 1283 informal caregivers and the 1283 people with dementia whom they provide care for from the cohort study. The results showed that lower QoL ratings by the person with dementia were associated with high caregiver stress, high perceived social restriction, and low caregiving competence.

Thus, the findings of this study highlighted the importance of developing targeted therapies and support systems by dementia healthcare professionals working in Kuwait. In clinical settings, such services could lessen the load on caregivers, advance their mental health, maintain their health, and improve overall care for both caregivers and their patients. Additionally, policymakers in Kuwait may need to prioritize investing in dementia-related support systems and educational services to empower caregivers and improve the quality of life for both caregivers and their patients.

This study showed that many confounders, such as gender, education level, employment status, and duration of care, had significant influences on caregivers. Many researchers supported these confounders. Abdelhalim et al. showed that the majority of the female participants were daughters who had a substantial burden in providing care and elevated levels of despair and anxiety. Additionally, Connors et al. [41] and Riffin et al. [42] showed that high levels of caregiver burden are present in a large proportion of female caregivers of people with dementia. This, aligned with our results, shows that caregiver gender appears to be the best predictor of this burden. This can create a significant burden, especially considering the lack of available professional support services. Such characteristics may help identify female caregivers at greater risk of burden to target for intervention.

Other variables associated with caregiving anxiety included education, employment status, and low level of perceived support [39]. Additionally, Kokorelias et al. [43] performed a review and showed that young-onset dementia affects individuals under 65 years of age, often leading to loss of employment and independence. Thus, family caregivers need support to obtain and sustain their occupations.

In addition, like the finding in this study, other researchers have concluded there is a relationship between the duration of caregiving for a person with dementia and burden, anxiety, and mental health well-being [44,45,46]. Noticeably, the result showed that approximately half of the CEWDs provided all necessary daily tasks to maintain the welfare of the family members with dementia. The never-ending support, care, and supervision, mainly when the duration of caring is more than one year, may place the caregiver under a significant amount of responsibility for the well-being of the family member with dementia, which can be a considerable source of burden and anxiety. With time and the progression of dementia, new complexities and challenges arise that may require additional physical, emotional, social, and psychological energy, determination, and resilience. In addition, more time may be devoted to caregiving for the elderly with dementia than to one’s own self-care, leisure, and social activities [47]. The continuous duration of caregiving and the imbalance between caregiving and self-care may exacerbate a diminished mental health well-being and further progress in the burden of care, anxiety, and depression. 

The overwhelming aspects of caring for an elderly person with dementia were reflected in acquiring more paid help (74%) and receiving more home healthcare support (75%) for CEWDs than CEWoDs (26% for paid help and 25% for home healthcare support). A basic purpose for seeking paid help is possibly to relieve some of the burden of care placed on the caregiver, to address the growing demands of the condition that may increase anxiety, and to allow an opportunity to take care of one’s well-being. Also, a primary reason for receiving home healthcare support is possibly due to the progression of the condition that may require specialized support, such as maintaining mobility, preserving daily functioning, and managing the condition, which the caregiver may be unable to provide. Win et al. [40] highlighted that a family member taking on the caregiving role before making adequate arrangements for a future career position, family welfare, financial security, and health conservation may experience a more significant burden and anxiety. Bjørge et al. [37] underlined that a caregiver can experience an intensified burden as their domestic tasks and caregiving roles may become an endless daily routine. Caregivers may develop a sense of entrapment, have limited participation in social activities, lack leisure time, and experience emotional instability.

This study shed light on the need for a comprehensive framework to fully understand the changing needs of caregivers and persons with dementia living in Kuwait as they move through their illness trajectory. It is well emphasized in the literature to provide a template to incorporate evidence-based research on understanding the value of relationships between caregivers and care recipients [48]. The Lazarus and Folkman model of coping [49] identified that there are two main coping strategies: (1) emotion-focused—e.g., avoidance, minimizing, seeking positive value in negative events—and (2) problem-focused—e.g., defining the problem, seeking alternative solutions, choosing, and acting. Studies found that using strategies such as problem-based coping, acceptance, and social–emotional support was helpful and beneficial for mental health and depression [50,51]. On the other hand, wishful thinking, denial, and avoidance strategies were associated with negative outcomes [50,52]. Thus, there is a need for health care settings in Kuwait dealing with dementia caregivers to focus more on psychoeducational interventions with the aim of developing problem-focused coping strategies.

### 4.1. Implications and Future Studies

Family caregivers of individuals with dementia may experience elevated levels of burden of care, anxiety, depression, and lower mental health well-being. Therefore, healthcare professionals should aim to improve the caregivers’ mental and physical health and well-being by offering family caregivers psychological and social support, educating them on the individual’s disease progression and phases, and preparing them for continuing changing behaviors [53,54,55]. Ultimately, by providing support, educational programs, and resources, the family caregiver can sustain caring for the family member with dementia for a longer time. Comprehensive management of the individuals with dementia must include building a partnership between healthcare professionals and family caregivers and implementing comprehensive psychosocial interventions. Moreover, support groups may provide a safe environment for caregivers to receive emotional support and exchange experiences and caring strategies. Finally, caregivers need to be educated on technology solutions for managing the care of the elderly with dementia, such as monitoring systems, smart home technology, safety alarms, tracking wearable devices, and medication management applications. 

Future studies may examine the effectiveness of intervention programs aimed at mitigating the burden, anxiety, and depression and improving mental health well-being. Future studies may include longitudinal studies that examine caregiving’s long-term effects on family caregivers, such as the burden of care, anxiety, depression, and mental health well-being. Also, we need to examine other predictive variables affecting dementia caregiving from the perspectives of Middle Eastern cultures and religions. This will provide a reliable understanding of caregivers’ burden, tolerance, and well-being. 

### 4.2. Limitations

This study had several limitations. The sample size was appropriate to reveal trends; however, a larger sample size should uncover more robust results. Participants were recruited via convenience sample, which affects external validity. The recruitment via social media application and hospital lists could limit generalizability to other caregivers. This study was also conducted in a specific region. Therefore, the generalization of the study findings is limited to that geographical location. Data on whether the presence of other chronic diseases affected CEWDs was not collected. Other factors not considered in this study include family structure, culture, religion, socioeconomics, adaptation strategies, party financing care, and the caregiver’s health status. 

## 5. Conclusions

Family caregivers of the elderly with dementia experienced moderate to severe burden of care, borderline anxiety and depression, and poor mental health well-being. The family caregivers experienced significant levels of burden of care and anxiety when caring for individuals with dementia more than the elderly without dementia. The duration of caregiving was a primary risk factor that may adversely affect the resilience of the caregiver. There is a need for researchers in Kuwait to participate in continued evidence-based knowledge and practice in the trajectory of dementia caregiving. Clinicians and policymakers need to provide further services related to dementia support systems in order to empower caregivers and improve the quality of life for both caregivers and their patients.

## Figures and Tables

**Table 1:** Demographic information of the participating caregivers for the elderly with and without dementia.

	CEWDs	CEWoDs	*p*-Value	Effect Size
Age			*p* < 0.001 **^a^	d = 0.48 medium
			*p* < 0.001 **^b^	r = 0.21 small
M ± SD	48.71 ± 10.74	42.81 ± 14.27		
N (%)	180 (65%)	98 (35%)		
Gender			*p* = 0.795 ^c^	ω = 0.016 small
Male	47 (26%)	27 (28%)		
Female	133 (74%)	71 (72%)		
Education			*p* = 0.034 *^c^	ω = 0.21 small
Less than HS	4 (02%)	9 (09%)		
High School	25 (14%)	20 (21%)		
Bachelor	122 (68%)	61 (62%)		
Graduate Studies	29 (16%)	8 (08%)		
Marital Status			*p* = 0.668 ^c^	ω = 0.075 small
Single	48 (27%)	33 (34%)		
Married	109 (60%)	53 (54%)		
Divorced	14 (08%)	7 (07%)		
Widowed	9 (05%)	5 (05%)		
Kin-relationship				
Daughter	126 (70%)	63 (64%)	*p* = 0.219 ^c^	ω = 0.13 small
Son	46 (26%)	27 (28%)		
Spouse	5 (03%)	2 (02%)		
Sister	3 (01%)	6 (06%)		
Brother	0 (00%)	0 (00%)		
Employment			*p* < 0.001 **^c^	ω = 0.30 medium
Employed	92 (51%)	46 (47%)		
Unemployed	11 (06%)	11 (11%)		
Retired	73 (41%)	26 (27%)		
Student	4 (02%)	15 (16%)		
Caring Duration			*p* = 0.490 ^c^	ω = 0.09 small
<1 Year	22 (12%)	18 (18%)		
1–5 Years	91 (51%)	43 (43%)		
6–10 years	53 (29%)	28 (28%)		
>10 Years	14 (08%)	09 (11%)		
Paid Help			*p* < 0.001 **^c^	ω = 0.23 small
Yes	124 (69%)	44 (45%)		
No	56 (31%)	54 (55%)		
Home Healthcare support			*p* < 0.001 **^c^	ω = 0.21 small
Yes	102 (57%)	34 (35%)		
No	78 (43%)	64 (65%)		
Caring Role			*p* = 0.028 *^c^	ω = 0.13 small
Some Tasks	93 (52%)	64 (65%)		
All Tasks	87 (48%)	34 (35%)		

^a^ Analyzed by a *t*-test; ^b^ analyzed by Mann–Whitney test; ^c^ analyzed by chi-square test; * significant at 0.05; ** significant at 0.001.

**Table 2:** Demographic information of the elderly (N = 278) with chronic health conditions and with and without dementia.

	Elderly with Dementia (n = 180, 65%)	Elderly Without Dementia (n = 98, 35%)	*p*-Value	Effect Size
Age			*p* < 0.001 **^a^	d = 0.50 medium
			*p* < 0.001 **^b^	r = 0.21 small
M ± SD	79.47 ± 8.13	75.24 ± 9.01		
N	180 (65%)	98 (35%)		
Gender			*p* = 0.866 ^c^	ω = 0.01 small
Male	57 (32%)	32 (33%)		
Female	123 (68%)	66 (67%)		
Marital Status			*p* = 0.010 *^c^	ω = 0.20 small
Single	23 (13%)	5 (5%)		
Married	86 (48%)	37 (38%)		
Divorced	15 (08%)	7 (7%)		
Widowed	56 (31%)	49 (50%)		

^a^ Analyzed by *t*-test of independent samples; ^b^ analyzed by Mann–Whitney test; ^c^ analyzed by chi-square test; * significance at 0.05; ** significance at 0.01.

**Table 3:** Descriptive statistics (mean, standard deviation, and levels) and *p*-values of the data collection scales between caregivers (N = 278) for the elderly with and without dementia.

Variable	CEWDs ^a^ (n = 180)	CEWoDs ^b^ (n = 98)	*p*-Value	Effect Size
Burden	µ = 17.21 ± 9.09 mdn = 16	µ = 14.51 ± 8.08 mdn = 13.5	*p* = 0.015 *^c^ *p* = 0.026 *^d^	d = 0.31 small r = 0.13 small
Levels	36 (20%) Mild	29 (29%) Mild	*p* = 0.034 *^e^	ω = 0.16 small
	76 (42%) Mod	46 (47%) Mod		
	68 (38%) Severe	23 (24%) Severe		
Well-being	µ 54.40 ± 25.10 mdn = 56	µ = 58.90 ± 23.42 mdn = 60	*p* = 0.145 ^c^ *p* = 0.133 ^d^	d = 0.18 small r = 0.09 small
Levels	19 (11%) High	12 (12%) High	*p* = 0.644 ^e^	ω = 0.05 small
	55 (31%) Mod	34 (35%) Mod		
	106 (59%) Low	52 (53%) Low		
Anxiety	µ 9.92 ± 5.15 mdn = 11	µ = 8.61 ± 4.79 mdn = 8	*p* = 0.039 *^c^ *p* = 0.027 *^d^	d = 0.26 small r = 0.13 small
Levels	51 (28%) Normal	44 (45%) Normal	*p* = 0.002 *^e^	ω = 0.22 small
	30 (17%) Borderline	22 (22%) Borderline		
	99 (55%) Abnormal	32 (33%) Abnormal		
Depression	µ 8.69 ± 4.35 mdn = 9	µ = 8.06 ± 4.24 mdn = 8	*p* = 0.247 ^c^ *p* = 0.130 ^d^	d = 0.15 small r = 0.09 small
Levels	53 (29%) Normal	41 (42%) Normal	*p* = 0.012 *^e^	ω = 0.18 small
	45 (25%) Borderline	30 (30%) Borderline		
	82 (46%) Abnormal	27 (28%) Abnormal		

^a^ Caregivers for the elderly with dementia; ^b^ caregivers for the elderly without dementia; ^c^ analyzed by the *t*-test of independent samples; ^d^ analyzed by the Mann–Whitney U test; ^e^ analyzed by the chi-square (X^2^) test; * significant at 0.05.

**Table 4:** Results of the ANOVA test of caregivers for the elderly with dementia (CEWDs, n = 180) and caregivers for the elderly without dementia (CEWoDs, n = 98) on the duration of caring.

CEWDs Group Caring Duration ^a^	<1 µ	1–5 µ	6–10 µ	>10 µ	*t*-Test	*p*-Value	Effect Size	Adjusted *p*-Value
ZBI-12	6.23	19.03	18	19.57	15.351	<0.001 **	0.207	<0.001 ** <1 and 1–5 <0.001 ** <1 and 6–10 <0.001 ** <1 and >10
Anxiety	7.14	10.42	10.38	9.36	2.685	0.048 *	0.044	0.044 * <1 and 1–5
Depression	6.72	9.25	8.36	10.07	3.485	0.017 *	0.056	0.022 * <1 and 1–5
WHO-5	72.1	50.1	56.5	46.0	5.595	0.001 *	0.087	0.001 * <1 and 1–5 0.011 * <1 and >10
**CEWoDs Group** **Caring Duration**	**<1** **µ**	**1–5** **µ**	**6–10** **µ**	**>10** **µ**	***t*-Test**	***p*-Value**	**Effect** **Size**	**Adjusted *p*-Value**
ZBI-12	9.44	17.0	13.1	17.1	4.809	0.004 *	0.133	0.004 * <1 and 1–5
Anxiety	6.39	9.81	7.93	9.44	2.596	0.057	0.077	---------------
Depression	5.67	8.98	7.64	9.78	3.387	0.021 *	0.098	0.030 * <1 and 1–5
WHO-5	65.7	59.63	57.1	47.11	1.359	0.260	0.042	---------------

^a^ Caring duration reported in years; * significant at <0.05; ** significant at <0.001.

## Data Availability

Data is contained within the article.

## References

[1] Chaaya M., Phung K., Atweh S., El Asmar K., Karam G., Khoury R., Ghandour L., Ghusn H., Assaad S., Prince M. (2017). Dementia and family burden of care in Lebanon. BJPsych Int..

[2] World Health Organization (2022). Ageing and Health. https://www.who.int/news-room/fact-sheets/detail/ageing-and-health.

[3] Prince M., Guerchet M., Prina M. (2015). The Epidemiology and Impact of Dementia—Current State and Future Trends. WHO Thematic Briefing.

[4] Alzheimer’s Association (2016). Alzheimer’s disease facts and figures. Alzheimer’s Dement..

[5] World Health Organization (2022). Dementia. https://www.who.int/news-room/fact-sheets/detail/dementia.

[6] Cerejeira J., Lagarto L., Mukaetova-Ladinska E.B. (2012). Behavioral and psychological symptoms of dementia. Front. Neurol..

[7] Alyafei A.H., Alqunaibet T., Mansour H., Ali A., Billings J. (2021). The experiences of family caregivers of people with severe mental illness in the Middle East: A systematic review and meta-synthesis of qualitative data. PLoS ONE.

[8] Pessotti C.F.C., Fonseca L.C., Tedrus G.M.A.S., Laloni D.T. (2018). Family caregivers of elderly with dementia Relationship between religiosity, resilience, quality of life and burden. Dement. Neuropsychol..

[9] Seidel D., Thyrian J.R. (2019). Burden of caring for people with dementia—Comparing family caregivers and professional caregivers. A descriptive study. J. Multidiscip. Healthc..

[10] Krutter S., Schaffler-Schaden D., Essl-Maurer R., Wurm L., Seymer A., Kriechmayr C., Mann E., Osterbrink J., Flamm M. (2020). Comparing perspectives of family caregivers and healthcare professionals regarding caregiver burden in dementia care: Results of a mixed methods study in a rural setting. Age Ageing.

[11] Peng H.L., Lorenz R.A., Chang Y.P. (2019). Factors associated with sleep in family caregivers of individuals with dementia. Perspect. Psychiatr. Care.

[12] Marvardi M., Mattioli P., Spazzafumo L., Mastriforti R., Rinaldi P., Polidori M.C., Cherubini A., Quartesan R., Bartorelli L., Bonaiuto S. (2005). The caregiver burden inventory in evaluating the burden of caregivers of elderly demented patients: Results from a multicenter study. Aging Clin. Exp. Res..

[13] Abreu W., Rodrigues T., Sequeira C., Pires R., Sanhudo A. (2018). The experience of psychological distress in family caregivers of people with dementia: A cross-sectional study. Perspect. Psychiatr. Care.

[14] Corey K.L., McCurry M.K. (2018). When Caregiving Ends: The experiences of former family caregivers of people with dementia. Gerontologist.

[15] Sheehan O.C., Haley W.E., Howard V.J., Huang J., Rhodes J.D., Roth D.L. (2020). Stress, burden, and well-being in dementia and nondementia caregivers: Insights from the caregiving transitions study. Gerontologist.

[16] Wang X., Cheng Z. (2020). Cross-Sectional Studies: Strengths, Weaknesses, and Recommendations. Chest..

[17] Kua E.H., Ko S.M. (1992). A questionnaire to screen for cognitive impairment among elderly people in developing countries. Acta Psychiatr. Scand..

[18] Faul F., Erdfelder E., Lang A.G., Buchner A. (2007). G*Power 3: A flexible statistical power analysis program for the social, behavioral, and biomedical sciences. Behav. Res. Methods.

[19] Zarit S.H., Reever K.E., Bach-Peterson J. (1980). Relatives of the impaired elderly: Correlates of feelings of burden. Gerontologist.

[20] Smith K.J., George C., Ferreira N., Haapala I., Biggs S., Kurrle S. (2018). Factors emerging from the “Zarit Burden Interview” and predictive variables in a U K sample of caregivers for people with dementia. Int. Psychogeriatr..

[21] Wang G., Cheng Q., Wang Y., Deng Y.L., Ren R.J., Xu W., Zeng J., Bai L., Chen S.D. (2008). The metric properties of Zarit caregiver burden scale: Validation study of a Chinese version. Alzheimer Dis. Assoc. Disord..

[22] Ojifinni O.O., Uchendu O.C. (2018). Validation and Reliability of the 12-item Zarit Burden Interview among Informal Caregivers of Elderly Persons in Nigeria. Arch. Basic Appl. Med..

[23] Bachner Y.G. (2013). Preliminary assessment of the psychometric properties of the abridged Arabic version of the Zarit burden interview among caregivers of cancer patients. Eur. J. Oncol. Nurs..

[24] Bjelland I., Dahl A.A., Haug T.T., Neckelmann D. (2002). The validity of the hospital anxiety and depression scale. An updated literature review. J. Psychosom. Res..

[25] Herrmann C. (1997). International experiences with the Hospital Anxiety and Depression Scale—A review of validation data and clinical results. J. Psychosom. Res..

[26] Zigmond A.S., Snaith R.P. (1983). The hospital anxiety and depression scale. Acta Psychiatr. Scand..

[27] Terkawi A.S., Tsang S., AlKahtani G.J., Al-Mousa S.H., Al Musaed S., AlZoraigi U.S., Alasfar E.M., Doais K.S., Abdulrahman A., Altirkawi K.A. (2017). Development and validation of Arabic version of the Hospital Anxiety and Depression Scale. Saudi J. Anaesth..

[28] Topp C.W., Østergaard S.D., Søndergaard S., Bech P. (2015). The WHO-5 Well-Being Index: A Systematic Review of the Literature. Psychother. Psychosom..

[29] Sischka P.E., Costa A.P., Steffgen G., Schmidt A.F. (2020). The WHO-5 well-being index—Validation based on item response theory and the analysis of measurement invariance across 35 countries. J. Affect. Disord. Rep..

[30] World Health Organization (1998). Well-Being measures in primary health care: The Dep-Care Project. Proceedings of the WHO Meeting.

[31] Serhatlioglu S., Yıldırım E. (2023). World Health Organization Well-being Index (WHO-5): A validation study on midwifery students and determination of related factors. Eur. J. Midwifery.

[32] Ohaeri J.U., Awadalla A.W. (2009). The reliability and validity of the short version of the WHO Quality of Life Instrument in an Arab general population. Ann. Saudi Med..

[33] IBM Corp. (2019). IBM SPSS Statistics for Windows, version 26.0.

[34] Moore D.S., McCabe G.P., Craig B.A. (2016). Introduction to the Practice of Statistics.

[35] American Psychological Association (2020). Publication Manual of the American Psychological Association.

[36] Abdelhalim D.S., Ahmed M.M., Hussein H.A., Khalaf O.O., Sarhan M.D. (2024). Burden of Care, Depression, and Anxiety Among Family Caregivers of People with Dementia. J. Prim. Care Community Health.

[37] Bjørge H., Sæteren B., Ulstein I.D. (2019). Experience of companionship among family caregivers of persons with dementia: A qualitative study. Dementia.

[38] Quinn C., Nelis S., Martyr A., Morris R., Victor C., Clareon L. (2020). Caregiver influences on ‘living well’ for people with dementia: Findings from the IDEAL study. Aging Ment. Health.

[39] Teahan Á., Lafferty A., Cullinan J., Fealy G., O’Shea E. (2021). An analysis of carer burden among family carers of people with and without dementia in Ireland. Int. Psychogeriatr..

[40] Win K.K., Chong M.S., Ali N., Chan M., Lim W.S. (2017). Burden among family caregivers of dementia in the oldest-old: An Exploratory Study. Front. Med..

[41] Connors M.H., Seeher K., Teixeira-Pinto A., Woodward M., Ames D., Brodaty H. (2020). Dementia and caregiver burden: A three-year longitudinal study. Int. J. Geriatr. Psychiatry.

[42] Riffin C., Van Ness P.H., Wolff J.L., Fried T. (2019). Multifactorial Examination of Caregiver Burden in a National Sample of Family and Unpaid Caregivers. J. Am. Geriatr. Soc..

[43] Kokorelias K.M., Li Z., Hitzig S.L. (2023). Understanding implementation characteristics in navigation programs for persons living with dementia and their caregivers: A scoping review. Int. J. Care Coord..

[44] La H.T., Hua C.L., Brown J.S. (2021). The Relationship Among Caregiving Duration, Paid Leave, and Caregiver Burden. Aging and the Family: Understanding Changes in Structural and Relationship Dynamics.

[45] Tulek Z., Baykal D., Erturk S., Bilgic B., Hanagasi H., Gurvit I.H. (2020). Caregiver Burden, Quality of Life and Related Factors in Family Caregivers of Dementia Patients in Turkey. Issues Ment. Health Nurs..

[46] Van den Kieboom R., Snaphaan L., Mark R., Bongers I. (2020). The Trajectory of Caregiver Burden and Risk Factors in Dementia Progression: A Systematic Review. J. Alzheimer’s Dis..

[47] Carvalho E.B., Neri A.L. (2019). Patterns of use of time by family caregivers of elderly persons with dementia. Rev. Bras. Geriatr. Gerontol..

[48] Gallagher-Thompson D., Choryan Bilbrey A., Apesoa-Varano E.C., Ghatak R., Kim K.K., Cothran F. (2020). Conceptual Framework to Guide Intervention Research Across the Trajectory of Dementia Caregiving. Gerontologist.

[49] Lazarus R.S., Folkman S. (1984). Stress, Appraisal and Coping.

[50] Gilhooly K.J., Gilhooly M.L.M., Sullivan M.P., McIntyre A., Wilson L., Harding E., Woodbridge R., Crutch S. (2016). A meta-review of stress, coping and interventions in dementia and dementia caregiving. BMC Geriatr..

[51] Li R., Cooper C., Austin A., Livingston G. (2013). Do changes in coping style explain the effectiveness of interventions for psychological morbidity in family carers of people with dementia?. A systematic review and meta-analysis. Int. Psychogeriatr..

[52] Li R., Cooper C., Bradley J., Shulman A., Livingston G. (2012). Coping strategies and psychological morbidity in family carers of people with dementia: A systematic review and meta-analysis. J. Affect. Disord..

[53] Steiner V., Pierce L.L., Salvador D. (2016). Information needs of family caregivers of people with dementia. Rehabil. Nurs..

[54] Sharif L., Basri S., Alsahafi F., Altaylouni M., Albugumi S., Banakhar M., Mahsoon A., Alasmee N., Wright R.J. (2020). An Exploration of Family Caregiver Experiences of Burden and Coping While Caring for People with Mental Disorders in Saudi Arabia—A Qualitative Study. Int. J. Environ. Res. Public Health.

[55] Rahmani F., Roshangar F., Gholizadeh L., Asghari E. (2022). Caregiver burden and the associated factors in the family caregivers of patients with schizophrenia. Nurs. Open.

